# Realising gene therapy: a biomedical challenge

**DOI:** 10.1016/j.ebiom.2024.105299

**Published:** 2024-08-14

**Authors:** eBioMedicine

In recent years, advancements in applications of gene therapy have accelerated. Gene therapy aims to treat and potentially cure people with genetic diseases that have a substantial effect on quality of life and for which no or few therapeutic options are available. As developments continue at a rapid pace, promising outcomes frequently feature prominently in the news. In November, 2023, the UK Medicines and Healthcare products Regulatory Agency gave the first conditional market authorisation of a CRISPR-based genome-editing therapy for the treatment of people with sickle cell disease and transfusion-dependent β-thalassaemia (TDT) for whom haematopoietic stem cell transplantation is appropriate but not available. The US Food and Drug Administration and the European Commission followed shortly thereafter. In the UK and EU, an estimated 10,000 people with sickle cell disease and TDT are potentially eligible for treatment.

These approvals were delivered after encouraging interim efficacy results from one clinical trial for each disease. The results, published in *The New England Journal of Medicine*, revealed that most people who received treatment remained free from severe vaso-occlusive crises in sickle cell disease or did not need red blood cell transfusion in TDT for at least 12 consecutive months. Sickle cell disease and TDT affect the ability of red blood cells to carry oxygen and are caused by genetic mutations in both copies of the *HBB* gene encoding haemoglobin. Rather than editing each mutation of individual people, the strategy of this treatment approach (known as exagamglogene autotemcel [exa-cel]) is to reinstate the expression of fetal globin genes, which are postnatally silenced by transcription factors such as BCL11A. More specifically, the enhancer regulating the expression of *BCL11A* is inactivated by ex-vivo genome-editing in bone-marrow stem cells, allowing them to express fetal haemoglobin. The cells are then transplanted back, where they can proliferate in the bone marrow.

In a study published in *The New England Journal of Medicine* in January, 2024, Longhurst and colleagues also used an indirect strategy for hereditary angioedema, thanks to which eligible participants could be administrated the same in-vivo CRISPR-based gene-editing therapy. Instead of editing the mutations in *SERPING1*, the gene causing the disease, the gene-editing strategy targeted the *KLKB1* gene encoding plasma kallikrein, a downstream protease in the dysregulated contact activation pathway. Ten participants received a single dose of the treatment. No severe adverse events were detected, plasma kallikrein concentrations were reduced for at least 24 weeks, and exploratory analyses promisingly showed a reduction in the number of angioedema attacks per month.

As discussed in a Review published in *The Lancet* in February, 2024, a core advantage of gene or genome-editing therapies over gene-addition therapies is its potential to expand the spectrum of targetable diseases to autosomal dominant genetic disorders, for which adding a non-mutated gene is not an adequate solution. Instead, genome-editing strategies can directly repair disease-causing mutations or modify gene or genome sequences. They also overcome a limitation of gene-addition therapies whereby the expression of the added gene, typically controlled by promoters and enhancers different from those found in nuclei of human cells, have little fine-tuning. Reflecting its versatility, therapeutic applications and ongoing clinical trials based on genome-editing strategies cited in this Review are as diverse as cancer immunotherapy, HIV infection, type 1 diabetes, or organ xenotransplantation.

Yet, the risk of unwanted effects exists and is linked to off-target editing (ie, unintended genetic modifications) and immunogenicity against the engineered nucleases, such as Cas9, or the viral-based vectors delivering the molecular components of this technology. Basic research and preclinical studies continue to provide computational and biological tools that help reduce this risk. For example, in a study published in *Molecular Therapy* in August, 2023, Stahl and colleagues optimised the transient delivery of Cas9 ribonucleoproteins in the mouse brain, leading to reduced immune responses but similar editing capacity in neurons to a commonly used adeno-associated virus (AAV)-based delivery.

Gene-addition therapies are complementary approaches to genome editing and, despite being used to treat human diseases for more than a decade, clinical trials continue to test their breadth of therapeutic applications. In a single-arm, single-centre trial published in *The Lancet* in May, 2024, Lv and colleagues used a gene-addition strategy to treat children who, due to biallelic mutations in the *OTOF* gene, had autosomal recessive deafness 9. A single injection of an AAV carrying a human *OTOF* transgene into the cochlea of six children aged 1–6 years with severe-to-complete hearing loss resulted in no dose-limiting toxicity or serious adverse events; five children had hearing recovery and improved speech perception.

However, successes in gene therapies should not make us forget disappointing outcomes. In June, 2024, Pfizer announced that boys aged 4–7 years with Duchenne muscular dystrophy treated with fordadistrogene movaparvovec, an AAV carrying a shortened version of the dystrophin gene, had no improvement of motor function 1 year after treatment compared with placebo. Besides no efficacy, safety concerns can also be a reason for poor outcomes. The ASPIRO trial evaluating the safety and efficacy of a single intravenous infusion of resamirigene bilparvovec was stopped after the death of four participants. The investigational AAV-based gene-replacement therapy of *MTM1* was administered to boys younger than 5 years who had X-linked myotubular myopathy (XLMTM) and required long-term mechanical ventilation. Exploratory analyses of the trial reported in *The Lancet Neurology* highlighted that undiagnosed cholestatic liver disease in children with XLMTM can potentially interact with the gene therapy, leading to serious hepatic and hepatobiliary adverse events and progressive liver disease unresponsive to immune suppression. Nevertheless, a promising reduction in ventilator dependence and improvement of motor function were observed in some surviving participants. In a companion Article published in *eBioMedicine*, Lawlor and colleagues reported a histopathological analysis of muscle biopsies showing improvement of organelle localisation and myofiber size 24 weeks after treatment.

Dowling and colleagues published in *Nature Medicine* in July, 2024, a study in which a single participant aged 4 years with hereditary spastic paraplegia type 50 was administrated an AAV-based gene-addition therapy of the target gene, *AP4M1,* intrathecally. After 12 months, no serious adverse events were detected and preliminary outcomes suggested that the progressive disease course, including developmental delay, microcephaly, and loss of motor skills, might be inhibited. Dowling and colleagues discussed time and costs as key aspects to consider when developing a single-gene therapeutic. In this case, development took almost 3 years from diagnosis to dosing and cost more than CA$3,500,000.

Affordability of treatment for all individuals in need remains an unsolved challenge in gene therapy. Although more than 75% of people with sickle cell disease are born in Africa, as reminded by Munung and colleagues in their review published in *Gene Therapy*, exa-cel trials have not involved any African countries so far. More generally, Munung and colleagues identified ethical, legal, and social issues that might need to be overcome for the implementation of gene therapy for people with sickle cell disease in Africa. Moreover, the scarce availability of genetic testing in many low-income and middle-income countries and the under-representation of diverse ethnic populations in genomic datasets and genetic research, impeding our understanding of genetic variants in many populations, are initial barriers keeping many individuals far from gene therapy.

As gene therapy enters the area of genome editing, a multidisciplinary and collaborative research effort is required to provide an efficient, safe, and affordable solution for all individuals with burdensome genetic disorders. In this field, basic and preclinical research have made and continue to make invaluable contributions to the clinic by deciphering biological underpinnings of diseases and providing the therapeutic tools to treat them. *eBioMedicine* welcomes translational research that addresses some of the challenges in this fundamentally different medical approach, in which a cure rather than a treatment might be achievable.

